# Inflammation in Liver Diseases

**DOI:** 10.1155/2018/3927134

**Published:** 2018-02-12

**Authors:** Dechun Feng, Partha Mukhopadhyay, Ju Qiu, Hua Wang

**Affiliations:** ^1^Laboratory of Liver Diseases, National Institute on Alcohol Abuse and Alcoholism, National Institutes of Health, Bethesda, MD 20892, USA; ^2^Laboratory of Cardiovascular Physiology and Tissue Injury, National Institute on Alcohol Abuse and Alcoholism, National Institutes of Health, Bethesda, MD 20892, USA; ^3^The Key Laboratory of Stem Cell Biology, Shanghai Institutes for Biological Sciences, University of Chinese Academy of Sciences, Chinese Academy of Sciences, Shanghai 200031, China; ^4^Anhui Medical University, Heifei 230032, China

The liver is the largest solid organ in our body responsible for nutrient metabolism and protein synthesis. Recently, accumulating evidence showed that the liver was not only a major metabolic organ but also an “immunologic organ.” Due to a strong and specific blood supply route through the liver, it maintains a unique immune microenvironment. Many liver-resident antigen-presenting cells also modulate immune regulatory function. Liver-resident Kupffer cells have an important role in phagocytosis to prevent invasion of pathogenic organisms from the intestine. Innate lymphocytes, including both natural killer cells and natural killer T cells, are enriched in the liver. The liver is also the major organ to produce acute-phase proteins which are closely associated inflammatory reactions.

The dysregulation of immune cells in the liver was critical in the pathogenesis of almost all types of liver diseases including viral hepatitis, autoimmune hepatitis, fatty liver, alcoholic liver diseases, cirrhosis, and drug/toxin-induced injury. The studies on liver inflammation will greatly improve the understanding of the mechanism of how liver immune cells interacted with hepatocytes and other cells in the liver to cause liver damage as well as liver repair after damage. These studies will also be very helpful in the development of a novel effective treatment for various liver diseases in the clinical setting.

In this special issue, we have invited a few papers that address such issues.

The first paper of this issue analyzed the change of etiology of liver cirrhosis patients in a hospital from 2002 to 2013 in China. They showed that the top four etiologies of cirrhosis were HBV, HCV, ALD, and autoimmune liver disease. The prevalence of HBV cirrhosis has decreased in the recent 12 years, which is related with the increased coverage of HBV vaccination in China. In contrast, alcoholic cirrhosis has increased significantly. The study supported the success of HBV vaccination in reducing HBV-related end-stage liver diseases and also suggested more attention should paid to alcoholic liver disease.

The second paper of this issue reviewed the role of IL-33, an IL-1 cytokine family member, in the pathogenesis of liver diseases. IL-33 functions as an alarmin that is released when barriers are breached. IL-33 binds to its receptor ST2 and activates NF-*κ*B, ERK, and JNK signaling. IL-33 is upregulated in fatty liver, and the treatment of IL-33 alleviated hepatic steatosis. In hepatitis, the role of IL-33 remains controversial. Most studies support IL-33 as protective in hepatitis; however, one report showed that the treatment of IL-33 exacerbated Con A-induced hepatitis. In addition, IL-33 is considered as a cytokine that can promote liver fibrosis in several animal models, and the levels of IL-33 are positively correlated with the severity of fibrosis in patients.

The third paper of this issue analyzed the prognostic value and causes of pretreatment liver injury in de novo diffuse large B-cell lymphoma (DLBCL) patients. Multivariate analysis revealed that liver dysfunction, advanced Ann Arbor stage, and elevated lactate dehydrogenase (LDH) were independent adverse prognostic factors of both progression-free survival (PFS) and overall survival (OS).

The fourth paper reviewed novel and important signaling molecules involved in the process of liver regeneration. The authors summarized cytokines in the priming phase; growth factors, Wnt signaling, and exosomes in the proliferation phase; and TGF-*β*/TGF-*β* family in the termination of liver regeneration. These information will be very helpful in understanding the mechanisms of liver regeneration and promoting liver repair after injury.

The fifth paper studied the protective effects of magnesium isoglycyrrhizinate (MgIG) in treating alcoholic liver disease. The authors used a novel chronic plus binge ethanol feeding-induced liver injury to evaluate the therapeutic effects of MgIG. MgIG significantly reduced the elevation of liver enzymes caused by alcohol and hepatic steatosis. The hepatoprotective effect of MgIG was associated with suppression of neutrophil ROS production as well as hepatocellular oxidative stress.

The sixth paper identified a novel biomarker cytokeratin 18 epitope M30 (M30 CK-18), which was correlated with liver inflammatory activity. M30 CK-18 can also discriminate patients with mild versus moderate-advanced fibrosis. The authors found that M30 CK-18 serum concentration had good sensitivity and specificity in discriminating mild versus moderate/severe fibrosis and inflammation even in patients with normal ALT activity.

The seventh paper investigated the role of hypoxia-inducible factor (HIF)1*α* in macrophage polarization. They found that overexpression of HIF1*α* in myeloid cells were in hyperinflammatory state characterized by the upregulation of M1 markers. Further metabolomics studies showed that HIF1*α* overexpression led to the increased glycolysis and pentose phosphate pathway intermediates which facilitated the M1 phenotype shift in macrophages.

*Dechun Feng* *Partha Mukhopadhyay* *Ju Qiu* *Hua Wang*

